# Underlying mechanisms involved in the decrease of milk secretion during *Escherichia coli* endotoxin induced mastitis in lactating mice

**DOI:** 10.1186/1297-9716-44-119

**Published:** 2013-12-05

**Authors:** Ken Kobayashi, Shoko Oyama, Takaaki Uejyo, Chinatsu Kuki, Md Morshedur Rahman, Haruto Kumura

**Affiliations:** 1 Laboratory of Dairy Food Science, Research Faculty of Agriculture, Hokkaido University, North 9, West 9, Sapporo 060-8589, Japan

## Abstract

Mastitis, the inflammation of mammary glands resulting from bacterial infection, disrupts milk production in lactating mammary glands. In this study, we injected lipopolysaccharide (LPS), one of the endotoxins from *Escherichia coli* into mouse mammary glands to disrupt milk production, and we investigated the influence of LPS on nutrient uptake, synthesis, and secretion processes for milk component production in alveolar epithelial cells (AEC). The expression of genes relevant to the three-staged milk component production process (nutrient uptake, synthesis, and secretion of milk components) were down-regulated within 12 h after LPS injection in AEC. The internalization of glucose transporter 1 (GLUT-1) from the basolateral membrane to the cytoplasm occurred in accordance with the down-regulation of gene expression 3 h after LPS injection. The abnormal localization of adipophilin and beta-casein was also observed in the LPS-injected mammary glands. SLC7A1, an amino acid transporter, was up-regulated 3 and 6 h after LPS injection. Furthermore, the inactivation of signal transducer and activator of transcription 5 (STAT5) and the activation of STAT3 and nuclear factor-kappa B (NFkappaB) occurred 3 h after LPS injection. These results indicate that the nutrient uptake, synthesis, and secretion of milk components in AEC are rapidly shut down in the lactating mammary glands after LPS injection.

## Introduction

Mammary glands supply milk as the sole nutrient source for suckling pups and maintain galactopoiesis during lactation. The milk contains abundant nutritive components such as caseins, lactose, and lipids. Such milk components are produced by the mammary alveolar epithelial cells (AEC). AEC take up nutrients from the blood stream as raw materials and synthesize several milk components in the subcellular organelles. Synthesized components are directionally secreted into the alveolar lumen through the apical membrane of AEC. Thus, AEC carry out milk component production through a three-staged process: nutrient uptake, synthesis, and secretion of milk components during lactation. However, mastitis, the inflammation of mammary glands resulting from bacterial infection, disrupts normal milk secretion from AEC [1]. Milk is the indispensable nutrient source for suckling offspring in mammals, and mastitis is the most costly common disease in the dairy industry. Therefore, it is important to know how infected bacteria inhibit milk component production through a three-staged process in AEC in mastitis.

At the first stage of milk component production, AEC take up glucose, glycerol, fatty acids, and amino acids as raw materials for milk component synthesis from the blood capillary network, which closely surrounds mammary alveoli [2]. To take up the enormous amount of raw materials, several types of transporters and channels exist in the basolateral membranes of AEC. Aquaporin 3 (AQP3) is a water and glycerol channel and localizes in the basolateral membrane of secretory AEC [3]. Glucose transporter 1 (GLUT-1) also exists in the basolateral membrane of ACE in lactating mammary glands [4]. The fatty acid transporter (solute carrier family 27, SLC27A) enhances the uptake of long-chain fatty acids into cells [5,6]. Neutral amino acid transporter 1 (ASCT1) and cationic amino acid transporter (CAT)-1, which are proteins encoded by the SLC1A4 and SLC7A1 genes, respectively, are expressed in porcine mammary glands during lactation [7].

At the second stage of milk component production, AEC synthesize milk components from raw materials. Milk-specific proteins such as caseins and whey acidic protein (WAP) are synthesized from amino acids stimulated by hormonal regulation via Signal Transducer and Activator of Transcription 5 (STAT5) activation during late pregnancy [8-11]. AEC also initiate the synthesis of lactose at the Golgi around the time of parturition [12]. Alpha-lactalbumin modifies the substrate specificity of UDP-glucosyltransferase and leads to the synthesis of lactose from glucose and UDP-galactose [13]. Triglycerides, the major lipid component of milk, are synthesized from glucose, glycerol, and fatty acids in or on the surface of the rough endoplasmic reticulum and are released into the cytoplasm as small cytoplasmic lipid droplets (CLD) [14]. The sterol regulatory element binding protein 1 (SREBP1) is a critical regulator of mammary secretory activation with regard to lipid biosynthesis [15,16]. Fatty acid binding protein 3 (FABP3) has also been reported to contribute to lipid biosynthesis by facilitating the intracellular transport of fatty acids [17]. These reports suggest that proteins, lactose, and lipids require gene expression relevant to their synthesis pathway in addition to raw materials.

At the final stage of milk component production, milk components are transferred to the apical membrane of AEC and are released into the alveolar lumen through specific intracellular pathways [18]. Caseins and lactose are released by exocytosis from the Golgi as secretory vesicles. Soluble *N*-ethylmaleimide-sensitive factor attachment protein receptor (SNARE) proteins regulate intracellular trafficking and exocytosis of secretory vesicles through the plasma membrane [19,20]. Specific SNARE proteins are predominantly arranged in distinct subcellular compartments and contribute to distinct trafficking pathways [21,22]. In lactating mammary glands, several SNARE proteins have been reported to be expressed in AEC, and SNAP-23, syntaxin-6, -7, and −12, as well as VAMP-4 and −8 have been suggested as candidate SNARE proteins involved in milk secretion in AEC [23]. Small CLD, which are synthesized in the rough endoplasmic reticulum, are released into the cytoplasm with a surface coat of proteins and polar lipids [24]. Small CLD fuse with each other and form larger CLD when being trafficked to the apical membrane [25]. Adipophilin has been identified as a major protein coating CLD and milk lipid globules (MLG), and the increase in the expression of adipophilin is correlated with the accumulation of CLD in AEC [15,26]. Adipophilin has also been suggested to function as an adaptor linking CLD to elements of the apical plasma membrane to facilitate their secretion with SNARE proteins [24,25,27]. Lipids exist in milk as MLG, which is a unique membrane-bound structure released in an apocrine fashion [28]. These reports suggest that some of the SNARE proteins and adipophilin are closely related with the exocytosis of secretory vesicles and the apocrine secretion of CLD.

AEC maintain nutrient uptake, synthesis, and secretion of milk components during lactation [29]. The normal milk production in lactating mammary glands is disrupted by mastitis, the inflammation of mammary glands resulting from bacterial infection [30,31]. However, it remains unclear how and when the three-staged milk component production process is inhibited in mastitis. In this study, we injected lipopolysaccharide (LPS), one of the endotoxins from *Escherichia coli* into mouse mammary glands to disrupt milk production because intramammary administration of LPS is a well-established method for experimental induction of mastitis under defined conditions to study the immune response of the mammary gland in cows [32-34]. AEC directly bind to LPS via the LPS-specific receptor, Toll-like receptor 4 [31,35]. We have previously reported that the rapid induction of apoptosis in AEC occurs immediately after LPS injection [36]. LPS weakens the milk-blood barrier by modulating claudins in AEC within 3 h after LPS treatment [37]. Thus, LPS adversely affects lactating AEC shortly after injection. Therefore, we investigated how and when AEC shut down galactopoiesis after LPS injection.

## Materials and methods

### Animals

Pregnant ICR mice were purchased from Japan SLC, Inc. (Shizuoka, Japan). After parturition, the lactating mice were kept with the suckling neonatal pups. LPS originating from *E. coli* 0111:B4 was solubilized in 0.5 mM CaCl_2_ and 0.5 mM MgCl_2_-containing phosphate-buffered saline (mPBS). LPS (20 μg) was injected into the fourth inguinal mammary gland via the teat canal without injury on day 10 of lactation under anesthesia with pentobarbital. Three, 6, or 12 h after LPS injection, the mice were decapitated, and the mammary glands were dissected. In addition, mice were removed from pups 30 min before dissection. In each of the experiments, the dissected mammary glands were washed with mPBS and then used immediately. In this study, we used non-treated mammary glands from non-treated mice as the control (0 h of LPS injection) because vehicle (mPBS)-injected mammary glands did not show any differences from mammary glands of mice without injection treatment [36,37]. All experimental procedures in this study were approved by the Animal Resource Committee of Hokkaido University and were conducted in accordance with the Hokkaido University guidelines for the care and use of laboratory animals.

### Materials

LPS was purchased from Sigma-Aldrich (St. Louis, MO). The following antibodies were used as primary antibodies for immunological studies: rabbit polyclonal antibodies against AQP3 (Alpha Diagnostic, San Antonio, TX), GLUT-1 (Millipore, Billerica, MA, USA), nuclear factor-kappa B (NFκB, Cell Signaling Technology, Danvers, MA, USA), phosphorylated-NFκB (Ser536, Cell Signaling), STAT3 (Santa Cruz Biotechnology, Santa Cruz, CA, USA), phosphorylated-STAT3 (pSTAT3, Tyr705, Cell Signaling), STAT5 (Cell Signaling), phosphorylated-STAT5 (pSTAT5, Tyr694, Cell Signaling), mouse monoclonal antibody against pan-keratin (Sigma-Aldrich), rat monoclonal antibody against CD11b (BioLegend, San Diego, CA, USA), goat polyclonal antibody against β-casein (Santa Cruz), and guinea pig polyclonal antibody against adipophilin (Cell Signaling). Secondary Alexa Fluor 488-conjugated goat anti-rabbit, anti-guinea pig, Alexa Fluor 546-conjugated goat anti-mouse, anti-rat, and Alexa Fluor 546-conjugated donkey anti-goat antibodies were purchased from Invitrogen/Molecular Probes (Eugene, OR, USA). The secondary horseradish peroxidase-conjugated anti-mouse, anti-rabbit, and anti-goat antibodies for western blotting analysis were purchased from Sigma-Aldrich.

### Quantitative real-time polymerase chain reaction

For quantitative real-time polymerase chain reaction (PCR), total RNA from the mammary glands was extracted using an RNeasy® Mini Kit (Qiagen, Valencia, CA, USA) according to the manufacturer’s instructions. Reverse transcription was performed using ReverTraAce® qPCR RT Master Mix (Toyobo, Osaka, Japan). Quantitative real-time PCR was performed in a Light Cycler® 480 (Roche Applied Science, Indianapolis, IN, USA) using the THUNDERBIRD® SYBR® qPCR Mix (Toyobo). The following outlines the amplification program: 95 °C for 1 min followed by 40 cycles at 95 °C for 15 s and 58 °C for 1 min. The information about primers is listed in Table 1. Glyceraldehyde-3-phosphate dehydrogenase (GAPDH) was used as an internal control.

**Table 1:** Primer sequences for real-time PCR in mouse mammary glands.

**Gene**	**Accession number**	**Primers**		**Product size**
		**Forward**	**Reverse**	
SLC1A4	NM_018861	cgcaggacagattttcacca	catccccttccacattcacc	197
SLC7A1	NM_007513	cgtccctcctcatttgcttc	gcgattacgggtgttttggt	289
SLC27A3	NM_011988	tctgggacgattgccagaaac	caagcgcaccttatggtcacac	116
AQP3	NM_016689	ctggacgctttcactgtgggc	gatctgctccttgtgtttcatg	309
GLUT-1	NM_011400	gcttcctgctcatcaatcgt	gccgaccctcttctttcatc	117
UGP2	NM_139297	tcacaaacaaaacacgagcaga	cacttgagcgatttccacca	89
PGM2	NM_028132	caagcaagctgtccctctgt	gatgtcctccacgctctgtt	137
α-lactalbumin	NM_010679	accagtggctacgacacac	cggggaactcactacttttacac	106
FABP3	NM_010174	agtcactggtgacgctggacg	aggcagcatggtgctgagctg	230
SREBP-1	NM_011480	gtcagcttgtggcagtggag	tctgagggtggaggggtaag	90
CSN1S1	NM_007784	cctttcccctttgggcttac	tgaggtggatggagaatgga	193
CSN2	NM_009972	cttcagaaggtgaatctcatggg	cagattagcaagactggcaagg	330
CSN3	NM_007786	tcgaccccattactcccattgtgt	tgtaaaaggtaagggaagacgagaaagat	289
WAP	NM_011709	aacattggtgttccgaaagc	agggttatcactggcactgg	179
Lactoferrin	NM_008522	ggctgagaaggcaggaaatg	tttggggctatggctaggtg	183
VAMP-3	NM_009498	gctgccactggcagtaatcgaagac	gagagcttctggtctctttc	113
VAMP-4	NM_016796	gggaccatctggaccaagatttgg	catccacgccaccacatttgcctt	225
Syntaxin-6	NM_021433	cgactggacaacgtgatgaa	ctgggcgaggaatgtaagtg	216
SNAP-23	NM_001177792	gtgttgtggcctctgcatct	ccatctcatcttctctggcatc	254
Adipophilin	NM_007408	caggggtggtggataagacc	ggtgataagcccgagagca	291
GAPDH	NM_008084	gagcgagaccccactaacatc	gcggagatgatgaccctttt	144

### Immunofluorescence staining

For immunofluorescence staining of paraffin sections (AQP3, pan-keratin, β-casein, adipophilin, and pSTAT3), the mammary glands were fixed with 4% formaldehyde in PBS, pH 7.4, for 1 day at 4 °C and embedded in paraffin. The embedded samples were sliced into 5-μm sections, and the sections were deparaffinized and hydrated. The sections were then immersed in antigen retrieval buffer (10 mM Tris and 0.5 mM EGTA in distilled water, pH 9.0) and heated in a microwave oven at 500 W for 20 min.

For immunofluorescence staining of frozen sections (GLUT-1, pan-keratin, CD11b, and NFκB), mammary glands were embedded in OCT compound (Tissue Tek, Sakura, Torrance, CA, USA) and rapidly frozen with liquid nitrogen. The frozen samples were sliced into 5-μm sections with a Leica CM 3050S cryomicrotome (Mannheim, Germany). The sections were fixed with 1% formaldehyde in PBS for 10 min at 4 °C and then with methanol for 10 min at −20 °C.

The paraffin-embedded and frozen sections were incubated with PBS containing 5% bovine serum albumin to block nonspecific interactions and were then treated with the respective primary antibody (diluted in blocking solution) overnight at 4 °C. After the sections were washed with PBS, they were exposed to the secondary antibody for 1 h at room temperature in blocking solution. Controls were treated in the same manner, except for the exclusion of the primary antibody. Images of the stained sections were obtained using a confocal laser-scanning microscope (TCS SP5; Leica) and LAS AF software (Leica).

### Western blotting analysis

The mammary glands were minced and lysed in buffer containing 1% Triton X-100, 1% SDS, 100 mM NaCl,10 mM HEPES (pH 7.4), 2 mM EDTA, a phosphatase inhibitor mixture (PhosStop, Roche), and a protease inhibitor mixture (complete mini, Roche). The lysates were then diluted with an equal volume of sample buffer (100 mMTris (pH 6.8), 100 mM dithiothreitol, 2% SDS, 0.2% bromophenol blue, and 20% glycerol), incubated for 10 min at 70 °C, and stored at −20 °C as samples for Western blotting.

The samples were electrophoresed using 8% or 12% sodium dodecyl sulfate-polyacrylamide gels and transferred onto polyvinylidene difluoride membranes (Bio-Rad, Hercules, CA, USA). The membranes were blocked for 1 h with PBS containing 4% nonfat dried milk and 0.05% Tween 20. In the case of β-casein, PBS containing 2% bovine serum albumin was used for blocking. The membranes were incubated overnight at 4 °C with primary antibodies diluted in PBS containing 2.5% bovine serum albumin. Subsequently, the membranes were washed in PBS containing 0.05% Tween 20 and incubated for 45 min at room temperature with the appropriate secondary horseradish peroxidase-conjugated antibodies diluted in the solutions used for blocking. The immunoreactive bands were detected using Luminate Forte Western HRP substrate (Millipore). The protein bands were visualized and quantified using a Bio-Rad ChemiDoc™ EQ densitometer.

### Measurement of lactose, triglycerides, and β-casein

Lactose, triglycerides, and β-casein, which were secreted and accumulated in mammary alveolar lumens and ducts as milk components, were extracted from the mammary glands of mice non-treated or treated with LPS. The minced mammary glands were suspended in 9 times its weight of PBS and then passed 30 times through a 19-gauge needle. The suspension was then centrifuged at 1500 × *g* for 5 min at room temperature. The supernatant (extract of accumulated milk in mammary alveolar lumens and ducts) was used for the measurement of lactose, triglycerides, and β-casein.

The measurement of lactose was performed using the F-kit for lactose/galactose (Boehringer Mannheim GmbH, Mannheim, Germany). Briefly, each extract was warmed at 70 °C for 15 min and mixed with 2 types of reaction buffers according to the manufacturer’s protocol. After incubation for 15 min at 25 °C, the absorbance of each mixture was measured at 340 nm.

The measurement of triglycerides was performed using the Adipogenesis Assay Kit (Biovision Inc., Mountain View, CA, USA) according to the manufacturer’s protocol. Briefly, each extract was warmed at 70 °C for 15 min and then diluted 9-fold with Lipid Extraction Solution. The diluted supernatant (50 μL) was transferred to each well of a 96-well assay plate followed by the addition of 50 μL of Assay Buffer and 2 μL of lipase solution to each well. After incubation for 10 min at 25 °C, 50 μL of the triglyceride reaction mixture was added to each well. The plate was incubated at 37 °C for 30 min, and the absorbance was read at 570 nm.

Beta-casein in the extract was measured by densitometry analysis of the β-casein bands detected by Western blotting. Each extract was vigorously vortexed and then diluted with 5 × Laemmli sample buffer for electrophoresis. After western blotting, densitometry analysis of the β-casein bands was performed using a Bio-Rad ChemiDoc™ EQ densitometer.

### Statistical analysis

Data are expressed as the mean (SD). The statistical significance of differences between the mean values was tested using a Student’s *t* test. Differences between the mammary glands of mice non-treated or treated with LPS were considered significant at a *p*-value < 0.05 and 0.005. All experiments were performed at least 4 times using different mice to ensure reproducibility.

## Results

### LPS down-regulates genes relevant for nutrient uptake

**Figure 1:**
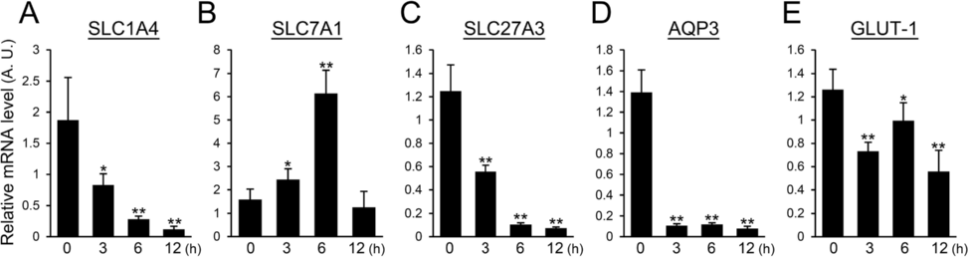
**Influence of LPS on the gene expression of transport proteins and channel proteins required to supply raw materials for milk.** Expression levels of SLC1A4 **(A)**, SLC7A1 **(B)**, SLC27A3 **(C)**, AQP3 **(D)**, and GLUT-1 **(E)** in mammary glands non-treated (0 h) and at 3, 6, and 12 h after LPS injection were quantified by real-time PCR. Data represent the mean (SD) (*n* = 6). *, *p* < 0.05; **, *p* < 0.005 vs. 0 h.

To evaluate the influences of LPS on the nutrient uptake process, we examined the expression levels of amino acid transporters (SLC1A4, SLC7A1), a fatty acid transporter (SLC27A3), a water and glycerol channel (AQP3), and a glucose transporter (GLUT-1) by real-time PCR. The expression level of SLC1A4 decreased by half at 3 h after LPS injection and was less than one-fifteenth of that observed in non-treated mammary glands after 12 h (Figure 1A). In contrast to SLC1A4, SLC7A1 showed a 4-fold increase in the expression level at 6 h after LPS injection and then returned to the expression level of non-treated mammary glands after 12 h (Figure 1B). The expression of SLC27A3 significantly declined by half at 3 h after LPS injection and was less than one-seventeenth of that seen in treated mammary glands at 12 h after LPS injection (Figure 1C). The expression level of AQP3 rapidly decreased after LPS injection to less than one-sixteenth of that observed in non-treated mammary glands and remained at the low expression level at 6 and 12 h after LPS injection (Figure 1D). The expression level of GLUT-1 significantly decreased approximately one-half at 3 h after LPS injection (Figure 1E).

**Figure 2:**
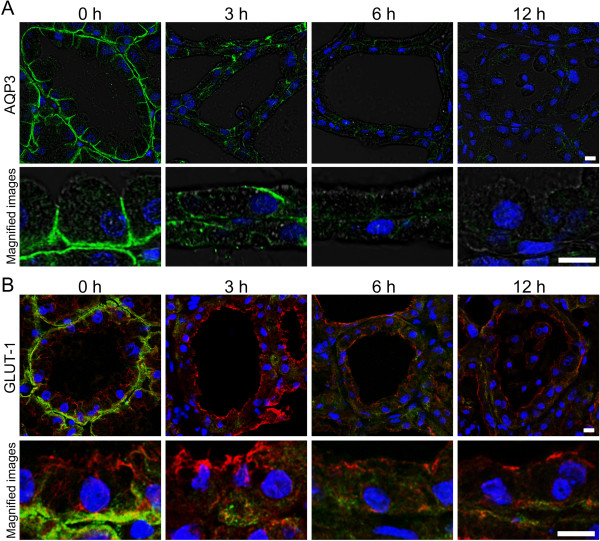
**LPS influence on the localization of AQP3 and GLUT-1 in AEC.** Mammary glands non-treated (0 h) and at 3, 6, and 12 h after LPS injection were immunostained with anti-AQP3 and anti-GLUT-1 antibodies. **(A)** Immunostained images of AQP3 (green) were merged with bright field images. **(B)** Immunostained images of GLUT-1 (green) were merged with pan-keratin (red) as a marker of epithelial cells. Blue represents nuclei stained with DAPI. Scale bars: 20 μm.

The localization patterns of AQP3 and GLUT-1 were examined by immunostaining. The localization of AQP3 was observed clearly in the basolateral membrane of AEC (Figure 2A). The staining intensity of AQP3 became weak after LPS injection after 3 h. GLUT-1 was localized in the basolateral membrane of AEC before LPS injection (Figure 2B). Three hours after LPS injection, the localization of GLUT-1 in the basolateral membrane was barely observed and GLUT-1 was sparsely localized to the basal sides of the cytoplasm. Six hours after LPS injection, GLUT-1 was localized across the cytoplasm but not on the plasma membrane. The localization of GLUT-1 partially returned to the basolateral membranes without obvious localization in the cytoplasm at 12 h after LPS injection.

### LPS down-regulates the genes relevant to the synthesis process

**Figure 3:**
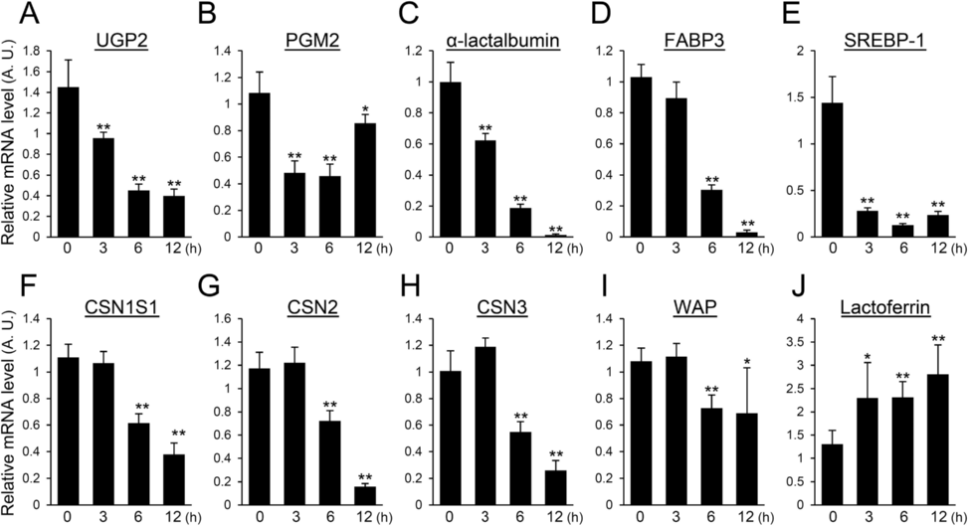
**Influence of LPS on the expression of genes related with milk component synthesis.** Expression levels of UGP2 **(A)**, PGM2 **(B)**, α-lactalbumin **(C)**, FABP3 **(D)**, SREBP-1 **(E)**, CSN1S1 **(F)**, CSN2 **(G)**, CSN3 **(H)**, WAP **(I)**, and lactoferrin **(J)** in mammary glands non-treated (0 h) and at 3, 6, and 12 h after LPS injection were quantified by real-time PCR. Data represent mean (SD) (*n* = 6). *, *p* < 0.05; **, *p* < 0.005 vs. 0 h.

The expression levels of genes related to the synthesis of milk components were determined by real-time PCR. UDP-glucose pyrophosphorylase 2 (UGP2), phosphoglucomutase 2 (PGM2), and α-lactalbumin participate in lactose synthesis [38]. The expression of UGP2 was gradually down-regulated and became less than one-fourth of the non-treated mammary glands at 12 h after LPS injection (Figure 3A). The expression level of PGM2 decreased by half at 3 h after LPS injection and was somewhat restored at 12 h after LPS injection (Figure 3B). The expression of α-lactalbumin was steadily down-regulated and became less than one-two hundredths of the non-treated mammary glands 12 h after LPS injection (Figure 3C). FABP3 and SREBP-1 are involved in the synthesis of triglycerides. FABP3 significantly decreased at 6 h after LPS injection, and the expression level of FABP3 at 12 h after LPS injection was less than one-thirtieth of that observed in non-treated mammary glands (Figure 3D). The level of SREBP-1 decreased significantly at 3 h after LPS injection and remained at a low expression level at 6 and 12 h after LPS injection (Figure 3E). The gene expressions of milk-specific proteins were also quantified. CSNS1, CSN2, and CSN3 decreased significantly at 6 h after LPS injection (Figure 3F-H). Twelve hours after LPS injection, the expression levels of CSNS1, CSN2, and CSN3 were approximately one-third, one-seventeenth, and one-fourth of those observed in non-treated mammary glands, respectively. WAP expression significantly declined at 6 h after LPS injection and was approximately two-thirds of that seen in the non-treated mammary glands (Figure 3I). Lactoferrin increased significanly after LPS injection to nearly twice that of non-treated mammary glands (Figure 3J).

### LPS partially down-regulates the expression of genes relevant to milk component trafficking

**Figure 4:**
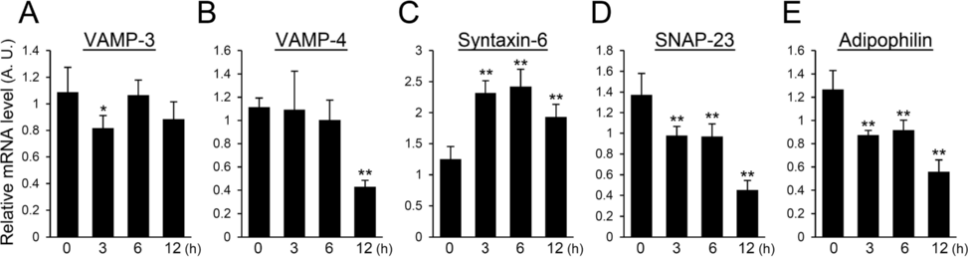
**Influence of LPS on the expression of genes required for milk component trafficking.** Expression levels of VAMP-3 **(A)**, VAMP-4 **(B)**, syntaxin-6 **(C)**, SNAP-23 **(D)**, and adipophilin **(E)** in mammary glands non-treated (0 h) and at 3, 6, and 12 h after LPS injection were quantified by real-time PCR. Data represent mean (SD) (*n* = 6). *, *p* < 0.05; **, *p* < 0.005 vs. 0 h.

To evaluate the influence of LPS on the release of synthesized milk components, the expression levels of SNARE, which take part in cellular membrane trafficking of transport vesicles, were quantified by real-time PCR. The expression level of VAMP-3 significantly decreased at 3 h after LPS injection but did not show a significant difference at 6 and 12 h after LPS injection (Figure 4A). VAMP-4 did not show a significant difference at 3 and 6 h after LPS injection but decreased to nearly one-third of that observed in non-treated mammary glands at 12 h after LPS injection (Figure 4B). The expression of syntaxin-6 in mammary glands treated with LPS increased approximately 2-fold compared to that of non-treated controls (Figure 4C). The expression level of SNAP-23 significantly decreased in mammary glands at 3 and 6 h after LPS injection and decreased to approximately one-third of that seen in non-treated mammary glands at 12 h after LPS injection (Figure 4D). We also investigated the expression level of adipophilin, which is involved in triglyceride storage and release. The adipophilin expression level declined after LPS injection similar to SNAP-23 (Figure 4E).

### LPS causes abnormal localization of milk components in AEC

**Figure 5:**
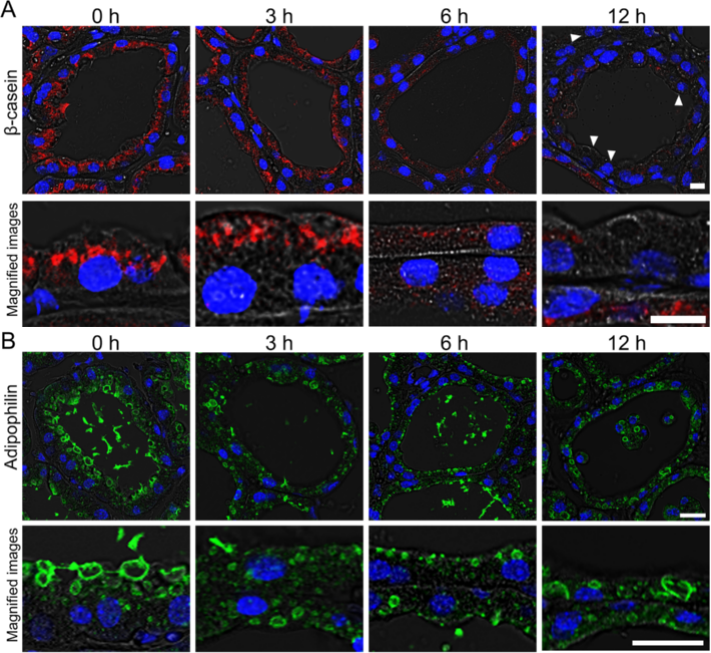
**Influence of LPS on the localization of β-casein and adipophilin in AEC.** Mammary glands non-treated (0 h) and at 3, 6, and 12 h after LPS injection were immunostained with anti-β-casein (red) and anti-adipophilin (green) antibodies. Immunostained images of β-casein **(A)** and adipophilin **(B)** were merged with bright field images and DAPI stained images (blue). Beta-casein-negative AEC were observed 12 h after LPS injection (arrowhead). Adipophilin staining shows the shape of lipid droplets in AEC. Scale bars: 20 μm.

The localizations of β-casein and adipophilin were investigated to determine the influence of LPS on intracellular trafficking of milk-specific proteins and CLD, respectively. Beta-casein was localized around the nuclei on the luminal side of the cytoplasm before LPS treatment (Figure 5A). At 3 h after LPS injection, β-casein was observed near the apical membrane. The staining intensity of β-casein became weak at 6 h after LPS injection. At 12 h after LPS injection, the staining intensity of β-casein was weak, and β-casein-negative AEC were also observed.

Adipophilin is a CLD-binding protein that covers the surface of CLD. Several sizes of CLD covered by adipophilin were observed in the cytoplasm of AEC in non-treated mammary glands (Figure 5B). The adipophilin-positive CLD became large toward the apical membrane of AEC. At 3 h after LPS injection, large adipophilin-positive CLD decreased throughout the cytoplasm of AEC. Adipophilin-positive CLD were hardly observed at 6 h after LPS injection. Large CLD reappeared at 12 h after LPS injection.

### LPS causes the decrease in milk components in mammary glands

**Figure 6:**
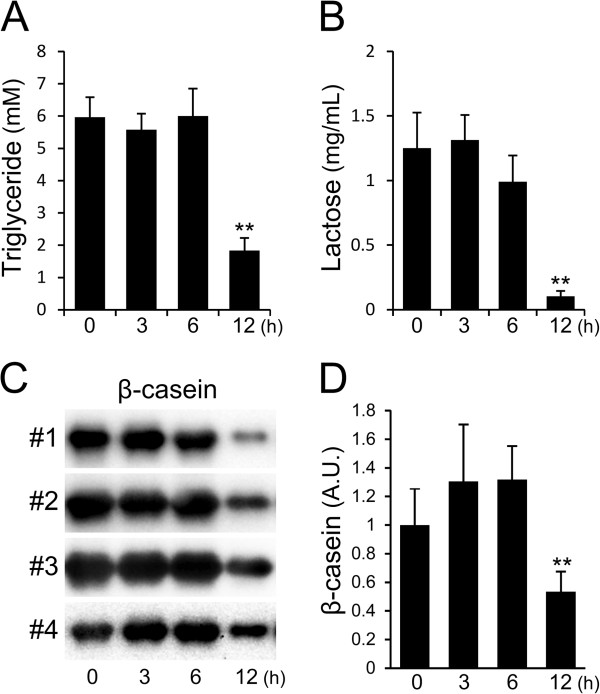
**Influence of LPS on the amount of milk components released into mammary glands.** The concentration of triglycerides **(A)** and lactose **(B)** in extracts from mammary glands non-treated (0 h) and at 3, 6, and 12 h after LPS injection were measured by the methods described in the Materials and Methods section. Beta-casein in the extracts was detected by western blotting **(C)**. The β-casein bands were quantified by densitometry analysis **(D)**. Data represent the mean (SD) (*n* = 8 for measuring triglycerides, *n* = 4 for measuring lactose, *n* = 6 for measuring β-casein). *, *p* < 0.05; **, *p* < 0.005 vs. 0 h.

To evaluate the influence of LPS on the release of major milk components, milk accumulated in the alveolar lumens and ducts was extracted from minced mammary glands and used for the determination of triglycerides, lactose, and β-casein contents. After 3 and 6 h, the concentration of triglycerides was approximately 6 mM in the extracts from LPS-treated and non-treated mammary glands (Figure 6A). A significant decrease in the triglyceride concentration was observed at 12 h after LPS injection; the concentration was reduced to less than 2 mM. The concentration of lactose was also decreased at 12 h after LPS injection, and the concentration was less than one-twelfth of that observed in non-treated mammary glands (Figure 6B). The protein level of β-casein in the extracts increased at 3 and 6 h after LPS injection and then decreased by half at 12 h after LPS injection compared to that of non-treated mammary glands (Figure 6C, D).

### LPS activates STAT3 and NFκB but inactivates STAT5

**Figure 7:**
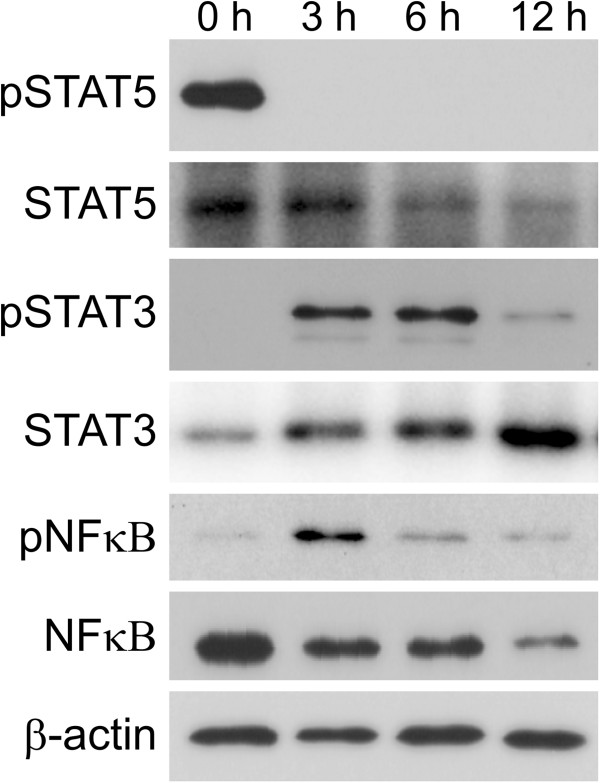
**Influence of LPS on STAT3, STAT5, and NFκB phosphorylation in mammary glands.** The phosphorylation of STAT3, STAT5, and NFκB were examined by western blotting analysis of mammary glands non-treated (0 h) and at 3, 6, and 12 h after LPS injection using antibodies against STAT5, phosphorylated STAT5, STAT3, phosphorylated STAT3, NFκB, and phosphorylated NFκB. Beta-actin was used as a control.

The STAT3 and NFκB pathways are activated by phosphorylation, and STAT5 is inactivated by dephosphorylation after weaning, which simultaneously causes the loss of milk production ability in AEC [39,40]. To confirm which signaling pathways are activated or deactivated by LPS treatment, the phosphorylation of STAT3, STAT5, and NFκB was examined. Phosphorylated STAT5 was detected in non-treated mammary glands but was not observed in mammary glands at 3, 6, and 12 h after LPS injection (Figure 7). In contrast, phosphorylated STAT3 was detected at 3 and 6 h after LPS injection and its level was decreased at 12 h after LPS injection. Phosphorylated NFκB was detected scarcely in non-treated mammary glands. An increase in phosphorylated NFκB was observed at 3 h after LPS injection. At 12 h after LPS injection, the levels of both NFκB and phosphorylated NFκB decreased.

**Figure 8:**
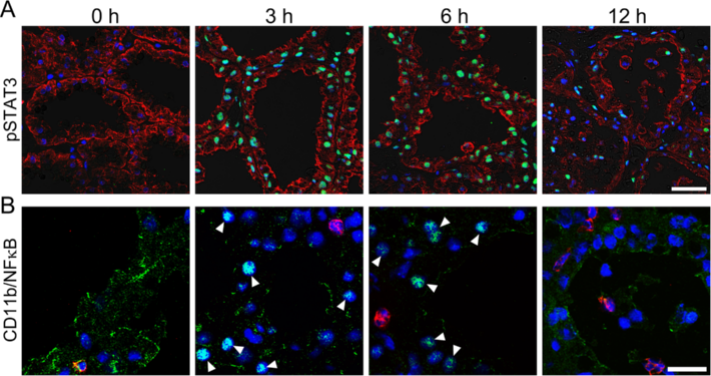
**LPS causes phosphorylation of STAT3, translocation of NFκB, and invasion of CD11b**^**+ **^**leucocytes into the mammary alveolar lumen.** Mammary glands non-treated (0 h) and at 3, 6, and 12 h after LPS injection were immunostained with anti-phosphorylated STAT3, anti-pan-keratin, anti-NFκB, and anti-CD11b antibodies. **(A)** Immunostained images of phosphorylated STAT3 (green) were merged with images of pan-keratin (red) as a marker of epithelial cells. **(B)** Immunostained images of NFκB (green) were merged with images of CD11b (red) as a marker of leucocytes containing neutrophils. Blue represents nuclear staining with DAPI. Arrowheads indicate the localization of NFκB in nuclei of AEC. Scale bars: 20 μm.

The activation of STAT3 and NFκB in AEC was detected by immunostaining. Phosphorylated STAT3 was localized in the nuclei of AEC at 3 h after LPS injection, although non-treated mammary glands did not show any positive reaction to phosphorylated STAT3 (Figure 8A). Phosphorylated STAT3 in the nuclei of AEC was also observed at 6 h after LPS injection, and some AEC showed phosphorylated STAT3 at 12 h after LPS injection. NFκB is known to be activated by phosphorylation in accordance with its translocation from the cytoplasm to the nucleus. The immunostaining of NFκB showed the translocation of NFκB from the cytoplasm to the nucleus in a portion of AEC at 3 and 6 h after LPS injection, whereas NFκB was observed in the cytoplasm but not in the nucleus at 12 h after LPS injection (Figure 8B). In addition, an immunoreaction to CD11b, which is a marker of leukocytes containing neutrophils, was observed in some cells localized in the alveolar lumen and interstitial tissues [41].

## Discussion

AEC express several lactation-specific genes to maintain milk component production during lactation [29]. However, it remains poorly understood how and when the milk component production shuts down after infection in mastitis. In this study, we categorized lactation-specific genes into the following 3 groups and investigated their temporal expression changes: nutrient uptake, synthesis, and secretion of milk components in AEC. The gene expressions of channels and transporters for the intake of water, glucose, glycerol, fatty acids, and amino acids were rapidly reduced by approximately half at 3 h after LPS injection. The immunostained images also revealed the decreases of GLUT-1 and AQP3 from the basolateral membranes of AEC at 3 h after LPS injection. These results indicate that the shutdown of the nutrient uptake occurs shortly after LPS injection. The expression levels of genes relevant to the synthesis of lactose and triglycerides declined by approximately half 3 h after LPS injection. The levels of 3 types of caseins and WAP also began to decrease at 6 h after LPS injection. The synthesis of milk components in AEC is also down-regulated within 6 h after LPS injection. Furthermore, the gene expressions for the secretion pathway and the exocytosis of milk components such as VAMP-4, SNAP-23, and adipophilin declined by approximately one-half were observed within 12 h after LPS treatment. Thus, LPS rapidly shut down the milk component production process, i.e., nutrient uptake, synthesis, and secretion of milk components in AEC.

Although the genes relevant to milk production are down-regulated in mastitis, lactoferrin and inflammatory cytokines such as interleukin (IL)-1β, IL-6, and tumor necrosis factor-α are up-regulated [42]. LPS also induces the rapid increase in mRNA for IL-1β, IL-8, and tumor necrosis factor-α after only 2 h in cultured bovine mammary epithelial cells [43]. In this study, lactoferrin was up-regulated after LPS injection although other genes relevant to the synthesis of milk components did not show up-regulation. Lactoferrin, which is released in infected tissues, is suggested to concern inflammation and immunomodulation processes in addition to its direct antimicrobial properties [44]. Thus, the up-regulation of inflammation-related proteins occurs simultaneously with the down-regulation of milk-specific proteins. In this study, SLC7A1, an amino acid transporter, and syntaxin-6, a SNARE protein, were upregulated. Interestingly, although the expression level of SLC1A4 is not changed, the mRNA level of SLC7A1 increases in mammary glands at peak lactation compared to early lactation, suggesting the presence of different regulatory mechanisms between the expression of SLC1A4 and SLC7A1 [45]. Furthermore, in mammary glands, there are several SNARE proteins whose relationship with lactation or cellular secretion of milk components remains unclear [23]. SLC7A1 and syntaxin-6 may contribute to the intake of amino acids and trafficking of inflammation-related proteins, respectively.

To initiate milk production, mammary glands require activation of STAT5 by prolactin [46]. In normal mammary glands, STAT5 is activated in termination of pregnancy and is rapidly inactivated after weaning, whereas the activation of STAT3 and NFκB is known to be key pathways involved in mammary gland involution [47]. Thus, the inactivation of STAT5 and the activation of STAT3 and NFκB represent the initiation of involution and the end of galactopoiesis. Interestingly, the inactivation of STAT5 and activation of NFκB and STAT3 in AEC also promotes milk loss in the mammary glands after infection [35,48]. Both STAT3 and NFκB have been shown to regulate the expression of genes involved in inflammation within the mammary glands in mastitis [49]. These reports have clearly shown the importance of STAT3, STAT5 and NFκB for shutdown of galactopoiesis in mastitis. However, it remains unclear how early the shutdown of galactopoiesis occurs in mastitis. Our study shows that the inactivation of STAT5 and activation of STAT3 and NFκB occurred at 3 h after LPS injection with down-regulation of genes relevant to the milk component production process and up-regulation of lactoferrin. AEC bind to LPS via Toll-like receptor 4, and the binding of LPS to Toll-like receptor 4 stimulates the activation of NFκB, recognized as translocation of NFκB from the cytoplasm to the nucleus [31]. Therefore, it is suggested that the rapid shutdown of galactopoiesis is induced through the binding of LPS to AEC and the activation of STAT3 and NFkB immediately after infection in mastitis. The recruitment of CD11b-positive leukocytes into the alveolar lumen, which is one of the symptoms in early mastitis, may occur after the shutdown of milk component production in AEC [1,41].

In this study, the down-regulation of lactation-specific genes relevant to nutrient uptake and synthesis of milk components occurred between 3 and 6 h after LPS injection. Immunostaining images of β-casein and adipophilin in AEC also revealed the posttranslational influences of LPS on the milk secretion process within 6 h after LPS injection. On the contrary, the amount of accumulated milk components (triglycerides, lactose, β-casein) in the alveolar lumens and ducts did not decrease until 12 h after LPS injection. These results suggest that LPS causes the abnormal accumulation of milk components in the alveolar lumens and ducts. We previously reported that myoepithelial cells maintain a contracted state at least for 12 h after LPS treatment [36]. The contraction of the smooth muscle in myoepithelial cells by oxytocin stimuli is required for milk ejection from the alveolar lumen [50]. The mice lacking smooth muscle actin are also unable to nurse their offspring [50,51]. LPS treatment may cause a defective milk ejection from alveolar lumen by myoepithelial cells in addition to the shutdown of galactopoiesis.

In summary, our results show that the expression of genes relevant to the three-staged milk component production process, consisting of nutrient uptake, synthesis, and secretion of milk components in AEC, are rapidly down-regulated in association with the inactivation of STAT5 and the activation of STAT3 and NFkB. Some of the proteins relevant to the milk component production process also show abnormal localization after LPS injection. Therefore, we suggest that lactating mammary glands rapidly shut down the milk production process after LPS injection through expressional control and posttranslational changes of proteins contributing to milk component production in AEC. However, several additional proteins and signaling pathways, which were not investigated in this study, have been reported to contribute to specific milk component production processes in AEC. Furthermore, AEC are surrounded by myoepithelial cells, microvascular endothelial cells, adipocytes, and several immune cell types such as macrophages and neutrophils. We previously reported that the cell-specific behavior and the tissue remodeling of the alveolus occur after LPS injection in accordance with disruption of the milk-blood barrier [36,37]. It is also suggested that the milk component production process is more intricately regulated.

## Competing interests

The authors declare that they have no competing interests.

## Authors’ contributions

KK: designed the study; analysis and interpretation of data; drafted the manuscript. SO, CK and TU: carried out the immunostaining studies and cell culture. RMM: interpreted data; revised the manuscript. HK: interpreted data; helped draft the manuscript; revised the manuscript. All authors read and approved the final manuscript.
